# Outcomes in diabetic macular edema switched directly or after a dexamethasone implant to a fluocinolone acetonide intravitreal implant following anti-VEGF treatment

**DOI:** 10.1007/s00592-019-01439-x

**Published:** 2019-11-20

**Authors:** Matus Rehak, Catharina Busch, Jan-Darius Unterlauft, Claudia Jochmann, Peter Wiedemann

**Affiliations:** Department of Ophthalmology, University Hospital Leipzig, Liebigstr. 10-14, 04103 Leipzig, Germany

**Keywords:** Diabetic macular edema, Fluocinolone acetonide, Dexamethasone, Second-line treatment, Best-corrected visual acuity, Central macular thickness

## Abstract

**Aims:**

Fluocinolone acetonide (FAc) is an intravitreal corticosteroid implant approved for the second-line treatment of diabetic macular edema (DME). This study compared outcomes of patients with DME switched directly to an FAc implant, versus indirectly via dexamethasone, after anti-VEGF therapy failure.

**Methods:**

This is a retrospective, single-center chart review. Patients were assigned to Group A (switched to FAc after anti-VEGF) or Group B (switched to dexamethasone and then to FAc after > 4 months). Charts were reviewed for best-corrected visual acuity (BCVA), central macular thickness (CMT), intraocular pressure (IOP) and cataract development.

**Results:**

Forty-nine eyes were included. BCVA increased and CMT decreased with anti-VEGF (both groups), and dexamethasone (Group B only), but regressed after stopping treatment. With FAc, BCVA increased rapidly and significantly: increases were maintained up to 36 months (*P* < 0.001), except at 18 and 9 months in Groups A and B, respectively. Significant CMT reductions (*P* < 0.001) were evident after 3 months and maintained up to 36 months in both groups. IOP increase > 21 mmHg occurred in 14 patients (nine in Group A, five in Group B): all were sufficiently treated with IOP-lowering drops. Nineteen phakic eyes (73.1%) developed cataract: seven underwent phaco-emulsification (two in Group A, five in Group B).

**Conclusions:**

Similar functional and anatomical improvements occurred in FAc-treated eyes, regardless of whether they first received dexamethasone or switched directly to FAc after anti-VEGF. Safety signals were consistent with corticosteroid class effects. Early switch to FAc could benefit patients who respond insufficiently to anti-VEGF.

**Electronic supplementary material:**

The online version of this article (10.1007/s00592-019-01439-x) contains supplementary material, which is available to authorized users.

## Introduction

Diabetic macular edema (DME) is a common complication of diabetic retinopathy, characterized by ischemic swelling of the central area of the retina, known as the macula [1]. Its prevalence in patients with diabetic retinopathy increases with the duration of diabetes and is estimated to be between 2.7% and 11% [2–4]. As population age and life expectancy increase, the prevalence of diabetes—and thus DME—is expected to rise [3].

DME significantly impacts quality of life. Functional ability declines with increasing visual impairment, and DME is the leading cause of vision loss among patients with diabetic retinopathy [2, 5]. Around two-thirds of people with DME are limited in their daily activities and those with diabetic eye diseases [1].

Without treatment, nearly half of those who develop DME will lose two or more lines of visual acuity within 2 years [6, 7]. Early diagnosis and effective treatment are therefore essential to avoid vision loss [5]. Current treatment guidelines for DME generally recommend anti-vascular endothelial growth factor (anti-VEGF) therapy as the first-line treatment and intravitreal corticosteroids as the second line [8–10].

The first-line intravitreal anti-VEGF injections have been shown in clinical trials to rapidly and effectively reduce the DME, and improve visual acuity and retinal morphology outcomes [11]. However, not all patients respond adequately: up to half show some persistent edema after anti-VEGF therapy alone [12].

For patients whose DME has responded sub-optimally to anti-VEGF therapy, treatment with intravitreal injections of corticosteroids has been shown to be beneficial [13–15]. Two implantable corticosteroid formulations are available in Europe: dexamethasone and fluocinolone acetonide (FAc). Both dexamethasone (OZURDEX^®^ 700 µg; Allergan Ltd, Marlow, Buckinghamshire, UK) and FAc (ILUVIEN^®^ 190 µg; Alimera Sciences Ltd, Aldershot, Hampshire, UK) are indicated for the treatment of vision impairment associated with DME that respond sub-optimally to available therapies [16, 17].

Both the dexamethasone and FAc implants are effective and have tolerable safety profiles for the treatment of DME [11, 18–21]. However, the FAc implant may offer improved convenience and a reduced clinical burden compared with the dexamethasone implant. While the latter is short acting (its effects lasting for around 6 months before re-injection is required), the FAc implant delivers inflammation-suppressing FAc for up to 3 years, with a daily release rate of 0.2 µg [11, 22].

It is unknown whether prior treatment with the short-acting dexamethasone implant after a sub-optimal response to anti-VEGF therapy influences outcomes in patients subsequently switched to the longer-acting FAc due to compliance or response concerns. Therefore, the objective of the current study was to compare the outcomes of patients with DME who switched to the FAc implant either directly, or indirectly (first to dexamethasone) following sub-optimal response to anti-VEGF therapy in clinical practice in a single center in Germany.

## Methods

### Study design

This was a retrospective, single-center chart review in patients with DME treated with an FAc intravitreal implant in a single center (University Hospital Leipzig) between February 1, 2014, and April 30, 2019. The study was conducted in accordance with the ethical principles of the Declaration of Helsinki [23], International Conference on Harmonization Good Clinical Practice Guidelines [24], and approved by an institutional review board.

### Patient population and study procedures

Patients with DME that have received FAc implant due to DME, which was non-responsive to previous anti-VEGF treatment with ranibizumab (given according to its approved license [25]), or which needed frequent re-treatment with ranibizumab were eligible for inclusion. Exclusion criteria included: patients with another concomitant ocular disease that could cause macular edema; patients with a history of previous pars plana vitrectomy; and previous use of intra- or periocular steroids.

Anti-VEGF-therapy was initiated in all eyes with three monthly ranibizumab injections followed by re-application using a pro re nata (PRN) regimen. A switch to intravitreal steroids was recommended in patients with sub-optimal response to anti-VEGF treatment, which was defined as a change in vision of ≤ 5 letters or a reduction of less than 20% in central macular thickness (CMT), measured using SD-OCT 1 month after the third intravitreal injection of ranibizumab. Further, patients requiring ranibizumab re-injection every 4 weeks after the 6 month of treatment were eligible to be switched to an intravitreal steroid.

Patients were divided into two subgroups: Group A, who were switched directly to an FAc implant after anti-VEGF therapy, or Group B, who were switched firstly to dexamethasone implants after anti-VEGF therapy and then to an FAc implant. Dexamethasone became available in Germany in October 2014, so the option to switch firstly to dexamethasone (Group B) following anti-VEGF therapy became available for the first time. Before this, FAc was the only option for patients who responded insufficiently on anti-VEGF therapy. Group B patients on dexamethasone were subsequently switched to FAc if they responded well to steroid treatment.

Both the dexamethasone implants (OZURDEX^®^, Allergan Ltd, Marlow, Bucks, UK) and FAc implants (ILUVIEN^®^, Alimera Sciences Ltd, Aldershot, Hants, UK) were injected singly into the affected eye of patients according to their licensed indications [17, 26].

### Outcomes

Outcomes were analyzed before the start of anti-VEGF therapy and 1 month after the last anti-VEGF injection (Groups A and B), before dexamethasone injection and 2 and > 4 months after (Group B only) and before FAc injection and 3, 6, 12, 18, 24, 30, and 36 months after (Groups A and B). Outcomes were also analyzed at the same time points for pseudophakic patients (Groups A and B). Efficacy evaluations were best-corrected visual acuity (BCVA) and central macular thickness (CMT). Safety was monitored in terms of mean change in intraocular pressure (IOP), the need for IOP-lowering eye drops and development of cataract.

### Data and statistical analysis

In the FAc group, the eyes were divided into three cohorts: those for which measurements were available for up to and including 12 months, 24 months and 36 months. These cohorts were not mutually exclusive of the others. A subgroup analysis was done for pseudophakic eyes. Student’s paired *t* tests were used to assess changes from pre-FAc baseline levels for Groups A and B and to establish significance between paired samples. A *P* value of < 0.05 was taken to indicate statistical significance. Data are reported as mean ± standard deviation (SD) unless otherwise stated. Statistical analysis was performed with SPSS Statistics 25 (IBM, Armonk, NY, USA).

## Results

### Patient population and demographics

Patient demographics and baseline characteristics (before first anti-VEGF injections) are shown in Table 1. A total of 49 eyes from 49 patients with DME were treated. Almost half (*n* = 23; 46.9%) of the eyes were pseudophakic. The mean duration of anti-VEGF treatment was 14.2 ± 10.3 months. During the period prior to the first FAc injection, macular or panretinal laser therapy was the most common additional treatment for DME or diabetic retinopathy (*n* = 21; 42.8% and *n* = 17; 34.7%, respectively).

**Table 1:** Patient demographics and baseline characteristics

	Group A (*n* = 24 eyes)	Group B (*n* = 25 eyes)	Overall cohort (*n* = 49 eyes)
*Before first anti*-*VEGF injection*			
Age, years, mean ± SD	61.8 (10.1)	62.2 (11.0)	62.0 (10.5)
Male, *n* (%)	17 (70.8)	13 (52.0)	30 (61.2)
Time since diagnosis of DME, months, mean ± SD (range)	7.38 ± 3.28 (2–14)	6.92 ± 3.46 (2–12)	7.14 ± 3.35 (2–14)
BCVA, ETDRS letters, mean ± SD	61.2 ± 13.1	64.0 ± 10.1	62.6 ± 10.1
CMT, µm, mean ± SD	546.8 ± 192.0	550.9 ± 74.8	548.9 ± 74.8
*Diabetes*			
Type 1, *n* (%)	2 (8.3)	1 (4.0)	3 (6.1)
Type 2, *n* (%)	22 (91.7)	24 (96.0)	46 (93.9)
HbA1c, %, mean ± SD	7.6 ± 1.2	7.5 ± 1.0	7.6 ± 1.1
Proliferative diabetic retinopathy, *n* (%)	5 (20.8)	4 (16.0)	9 (18.4)
Glaucoma, *n* (%)	3 (12.5)	9 (36.0)	12 (24.5)
Pseudophakic, *n* (%)	11 (45.8)	12 (48.0)	23 (46.9)
*Therapies for diabetic retinopathy or DME until FAc treatment*			
Anti-VEGF, *n* (%)	24 (100.0)	25 (100.0)	49 (100.0)
Number of anti-VEGF injections, mean ± SD	8.37 (5.0)	5.1 (2.5)	6.74 (6.2)
Treatment duration, months, mean ± SD	16.5 (11.8)	10.4 (8.2)	14.2 (10.3)
Macular laser therapy, *n* (%)	12 (50.0)	9 (24.0)	21 (42.8)
Panretinal photocoagulation, *n* (%)	9 (37.5)	8 (32.0)	17 (34.7)
Dexamethasone, *n* (%)	0.0	25 (100.0)	–
Number of dexamethasone implants, mean ± SD	0.0	1.27 (0.14)	–
Treatment duration, months, mean ± SD	0.0	6.5 (2.7)	–

*BCVA* best-corrected visual acuity, *CMT* central macular thickness, *DME* diabetic macular edema, *ETDRS* early treatment diabetic retinopathy study, *HbA1c* glycated hemoglobin, *SD* standard deviation, *VEGF* vascular endothelial growth factor

No statistical differences in the baseline parameters (Table 1) and the follow-up time were found between Groups A and B (*P* > 0.05 for all baseline characteristics). Patients in Group A were followed up for a median 31.6 ± 12.3 (12–48) months, and patients in Group B were followed up for a median 25.3 ± 8.5 (12–38) months.

## Functional and anatomical outcomes

### Group A

**Fig. 1:**
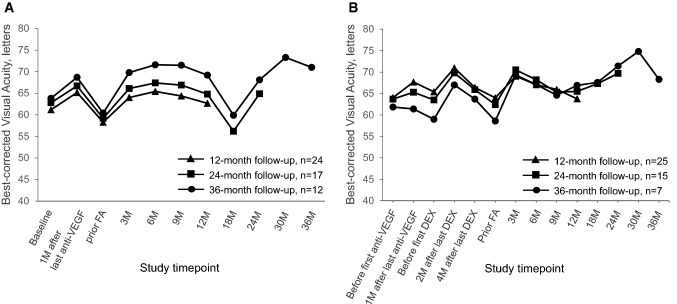
Mean change in BCVA in patients switched either directly (Group A; Panel A) or indirectly (Group B; Panel B) from anti-VEGF therapy to FAc implant. *DEX* dexamethasone, *ETDRS* early treatment diabetic retinopathy study, *FAc* fluocinolone acetonide, *VEGF* vascular endothelial growth factor

**Fig. 2:**
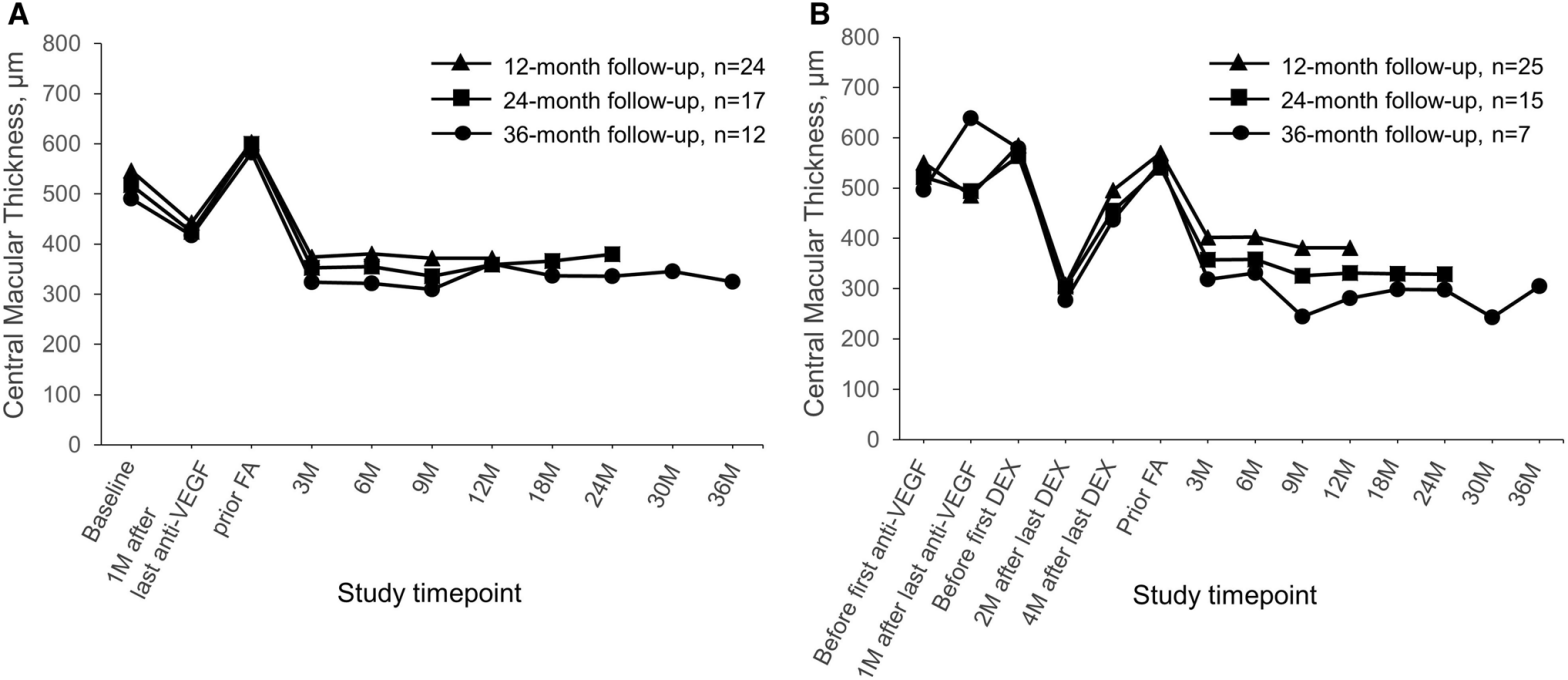
Mean change in CMT in patients switched either directly (Group A; Panel A) or indirectly (Group B; Panel B) from anti-VEGF therapy to FAc implant. *CMT* central macular thickness, *DEX* dexamethasone *FAc* fluocinolone acetonide, *VEGF* vascular endothelial growth factor

During anti-VEGF therapy, BCVA increased and CMT decreased in all three cohorts in Group A (Figs. 1A, 2A, Tables 2, 3). However, after discontinuation of anti-VEGF therapy, BCVA and CMT worsened, and before FAc injection values below those at baseline were reached (overall Group A: − 2.9 ± 7.8 letters, + 56.7 ± 176.4 µm).

**Table 2:** BCVA at each study time point in patients switched directly (Group A) or indirectly (Group B) from anti-VEGF therapy to FAc implant

BCVA at each study time point, ETDRS letters	Group A			Group B		
	12-month cohort (*n* = 24)	24-month cohort (*n* = 17)	36-month cohort (*n* = 12)	12-month cohort (*n* = 25)	24-month cohort (*n* = 15)	36-month cohort (*n* = 7)
Before anti-VEGF	61.2 (13.1	62.8 (13.2)	63.8 (12.2)	64.0 (10.1)	63.7 (9.2)	61.9 (11.5)
After anti-VEGF *P* value versus pre-VEGF	65.2 (11.9) **0.028**	66.7 (12.0) 0.071	68.7 (12.0) **0.028**	67.7 (12.3) 0.055	65.3 (13.9) 0.461	61.4 (18.1) 0.92
Before DEX	–	–	–	65.4 (9.7)	63.5 (11.2)	59.0 (14.2)
2 months after DEX *P* value versus pre-anti-VEGF	–	–	–	71.0 (10.3) **< 0.001**	69.8 (10.9) **0.081**	67.0 (13.0) **< 0.001**
4 months after DEX *P* value versus pre-anti-VEGF	–	–	–	66.4 (11.3) 0.131	65.9 (12.5) 0.207	63.7 (15.3) 0.386
Before FAc	58.3 (12.2)	59.2 (11.2)	60.4 (9.9)	64.0 (9.7)	62.4 (10.6)	58.6 (12.1)
3 months after FAc *P* value versus pre-FAc	64.0 (14.4) **< 0.001**	66.1 (12.7) **< 0.001**	69.8 (9.7) **< 0.001**	69.0 (11.3) **< 0.001**	70.5 (11.4) **< 0.001**	69.3 (13.8) **< 0.001**
6 months after FAc *P* value versus pre-FAc	65.4 (13.1) **< 0.001**	67.4 (12.1) **< 0.001**	71.6 (10.2) **< 0.001**	67.0 (10.7) 0.055	68.2 (11.9) **0.003**	67.1 (14.3) **< 0.001**
9 months after FAc *P* value versus pre-FAc	64.4 (14.2) **< 0.001**	66.9 (13.5) **< 0.001**	71.5 (9.9) **< 0.001**	66.0 (14.5) 0.366	65.3 (17.2) 0.342	64.6 (15.6) 0.061
12 months after FAc *P* value versus pre-FAc	62.7 (15.4) 0.059	64.8 (13.9) **0.046**	69.2 (12.0) **0.006**	63.8 (15.1) 0.928	65.5 (17.0) 0.378	66.9 (17.1) **0.015**
18 months after FAc *P* value versus pre-FAc	– –	56.2 (19.9) 0.478	59.9 (18.4) 0.925	– –	67.3 (13.3) 0.125	67.6 (14.3) **< 0.001**
24 months after FAc P value versus pre-FAc	– –	64.9 (13.6) 0.093	68.1 (14.2) 0.082	– –	69.7 (11.4) **0.011**	71.4 (12.7) **< 0.001**
30 months after FAc *P* value versus pre-FAc	– –	– –	73.3 (5.7) **< 0.001**	– –	– –	74.8 (7.6) **< 0.001**
36 months after FAc *P* value versus pre-FAc	– –	– –	71.0 (8.8) **< 0.001**	– –	– –	68.3 (12.2) **< 0.001**

All values are mean (SD). Significant values are shown in bold

*BCVA* best-corrected visual acuity, *DEX* dexamethasone, *ETDRS* early treatment diabetic retinopathy study, *FAc* fluocinolone acetonide, *VEGF* vascular endothelial growth factor

**Table 3:** CMT at each study time point in patients switched directly (Group A) or indirectly (Group B) from anti-VEGF therapy to FAc implant

CMT at each study time point, µm	Group A			Group B		
	12-month cohort (*n* = 24)	24-month cohort (*n* = 17)	36-month cohort (*n* = 12)	12-month cohort (*n* = 25)	24-month cohort (*n* = 15)	36-month cohort (*n* = 7)
Before anti-VEGF	546.8 (192.0)	517.4 (177.4)	490.8 (161.9)	550.9 (74.8)	521.8 (70.0)	497.0 (68.3)
After anti-VEGF *P* value versus pre-VEGF	442.5 (172.8) **0.01**	424.0 (191.8) 0.076	417.2 (179.8) 0.119	485.4 (165.4) 0.074	494.2 (186.5) 0.604	639.3 (151.7) **0.015**
Before DEX	–	–	–	584.7 (110.8)	563.7 (119.5)	579.3 (154.6)
2 months after DEX *P* value versus pre-anti-VEGF	–	–	–	308.1 (83.9) **< 0.001**	304.3 (81.2) **< 0.001**	277.1 (91.9 **< 0.001**
4 months after DEX *P* value versus pre-anti-VEGF	–	–	–	495.5 (163.1) 0.069	455.1 (158.6) 0.069	437.1 (207.3) 0.31
Before FAc	603.4 (166.6)	600 (167.8)	582.7 (184.1)	569.6 (127.0)	540.1 (110.0)	550.6 (138.0)
3 months after FAc *P* value versus pre-FAc	374.3 (144.9) **< 0.001**	352.3 (145.3) **< 0.001**	323.7 (103.3) **< 0.001**	402.0 (138.2) **< 0.001**	357.2 (119.4) **< 0.001**	318.0 (115.6) **< 0.001**
6 months after FAc *P* value versus pre-FAc	380.6 (154.9) **< 0.001**	355.0 (160.6) **< 0.001**	321.8 (142.6) **< 0.001**	402.9 (149.8) **< 0.001**	358.3 (121.5) **< 0.001**	330.9 (137.9) **< 0.001**
9 months after FAc *P* value versus pre-FAc	372.0 (144.0) **< 0.001**	336.1 (122.1) **< 0.001**	309.3 (101.3) **< 0.001**	381.1 (178.1) **< 0.001**	325.6 (143.1) **< 0.001**	244.7 (71.4) **< 0.001**
12 months after FAc *P* value versus pre-FAc	371.6 (143.1) **< 0.001**	359.4 (146.5) **< 0.001**	360.6 (165.6) **< 0.001**	381.0 (179.1) **< 0.001**	330.9 (130.1) **< 0.001**	281.3 (96.1) **< 0.001**
18 months after FAc *P* value versus pre-FAc	– –	365.9 (165.3) **< 0.001**	336.1 (134.1) **< 0.001**	– –	329.2 (113.8) **< 0.001**	298.4 (125.1) **< 0.001**
24 months after FAc *P* value versus pre-FAc	– –	379.5 (173.4) **< 0.001**	336.1 (130.3) **< 0.001**	– –	328.4 (139.8) **< 0.001**	297.7 (151.1) **< 0.001**
30 months after FAc *P* value versus pre-FAc	– –	– –	345.3 (153.8) **< 0.001**	– –	– –	243.2 (64.1) **< 0.001**
36 months after FAc *P* value versus pre-FAc	– –	– –	325.1 (147.0) **< 0.001**	– –	– –	304.7 (136.1) **< 0.001**

All values are mean (SD). Significant values are shown in bold

*CMT* central macular thickness, *DEX* dexamethasone. *FAc* fluocinolone acetonide, *VEGF* vascular endothelial growth factor

In the first 12 months after FAc injection, functional and anatomical outcomes improved in all three subgroups (Figs. 1A, 2A, Table 2, 3). However, BCVA dropped at Month 18 (Month 12 vs Month 18: 24-month cohort, − 8.6 ± 9.2 letters; 36-month cohort, − 9.3 ± 10.8 letters), but CMT remained stable (Month 12 vs Month 18: 24-month cohort, + 6.5 ± 107.0 µm; 36-month cohort, − 24.5 ± 62.6 µm). By the end of the follow-up, BCVA had improved by 4.4 letters (*P* = 0.059), 5.7 letters (*P* = 0.093) and 10.6 letters (*P* < 0.001), and CMT was reduced by 231.8 µm, 220.5 µm and 257.6 µm (all *P* < 0.001) in the 12-, 24- and 36-month cohorts, respectively.

### Group B

In Group B (Figs. 1B, 2B, Tables 2, 3), anti-VEGF therapy induced a BCVA gain of + 3.7 ± 9.5 letters and a CMT reduction of 65.5 ± 183.5 µm (overall Group B). However, in the 36-month cohort, BCVA changed by − 0.5 ± 6.0 letters and CMT increased by 142.5 ± 103.7 µm. At the time of dexamethasone implantation, anatomical and functional values were similar to those before anti-VEGF therapy (overall Group B, baseline vs prior dexamethasone implant: BCVA: + 1.4 ± 9.1 letters, *P* = 0.455; CMT: + 33.8 ± 114.5 µm, *P* = 0.141). The dexamethasone implant led to a BCVA improvement of + 5.6 ± 6.5 letters and + 1.0 ± 7.5 letters after 2 and 4 months, respectively (overall Group B). However, at the time of the switch to FAc, anatomical and functional values were again similar to those at baseline (prior anti-VEGF therapy, BCVA: ± 0.0 ± 7.8 letters, *P* = 0.98; CMT: + 18.7 ± 123.0, *P* = 0.45). Three months after FAc injections, BCVA and CMT improved significantly by + 5.0 ± 6.5 letters and − 167.6 ± 132.0 µm, respectively *P* < 0.001). By the end of the follow-up, BCVA changed by − 0.2 letters (*P* = 0.928), + 7.3 letters (*P* = 0.011) and + 9.7 letters (*P* < 0.001), and CMT was significantly reduced by − 188.6 µm, − 211.7 µm and − 245.6 µm (all *P* < 0.001) in the 12-, 24- and 36-month cohorts, respectively.

### Pseudophakic eyes

**Fig. 3:**
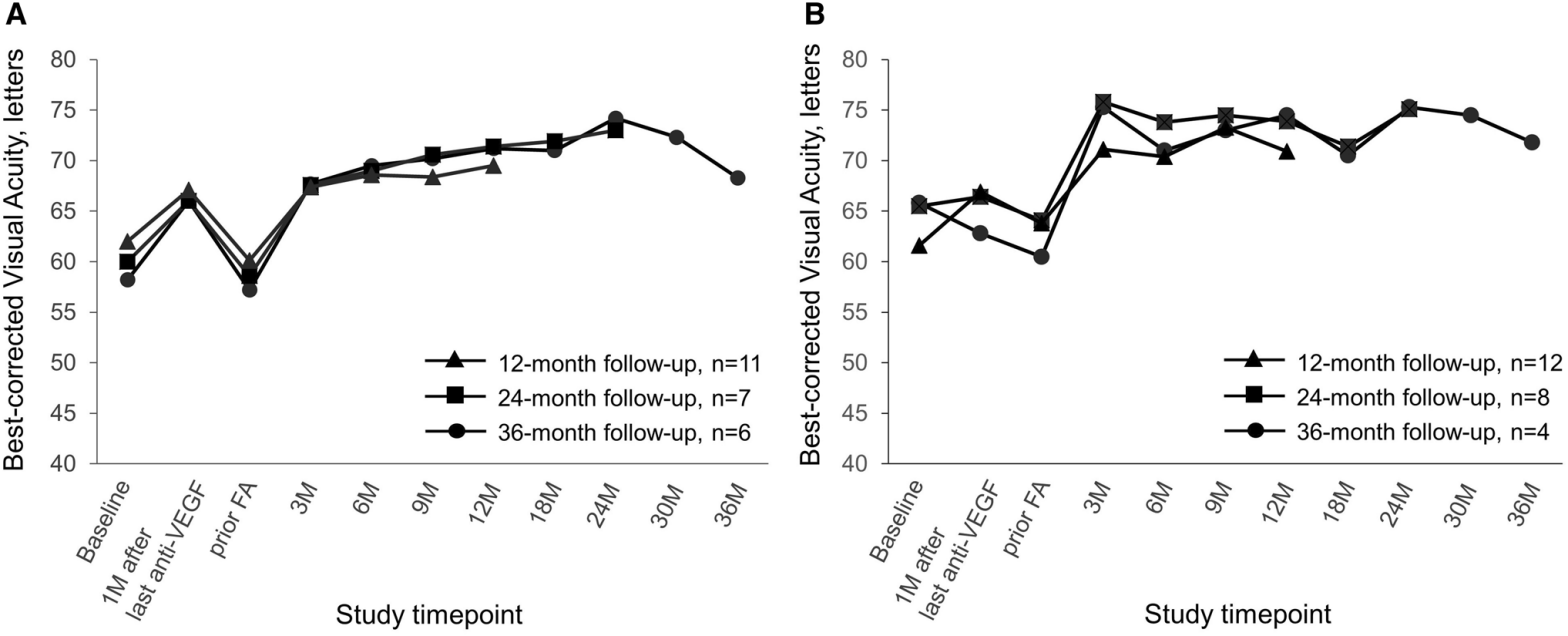
Mean change in BCVA in pseudophakic eyes switched either directly (Group A; Panel A) or indirectly (Group B; Panel B) from anti-VEGF therapy to FAc implant. *BCVA* best-corrected visual acuity, *ETDRS* early treatment diabetic retinopathy study, *FAc* fluocinolone acetonide, *VEGF* vascular endothelial growth factor

**Fig. 4:**
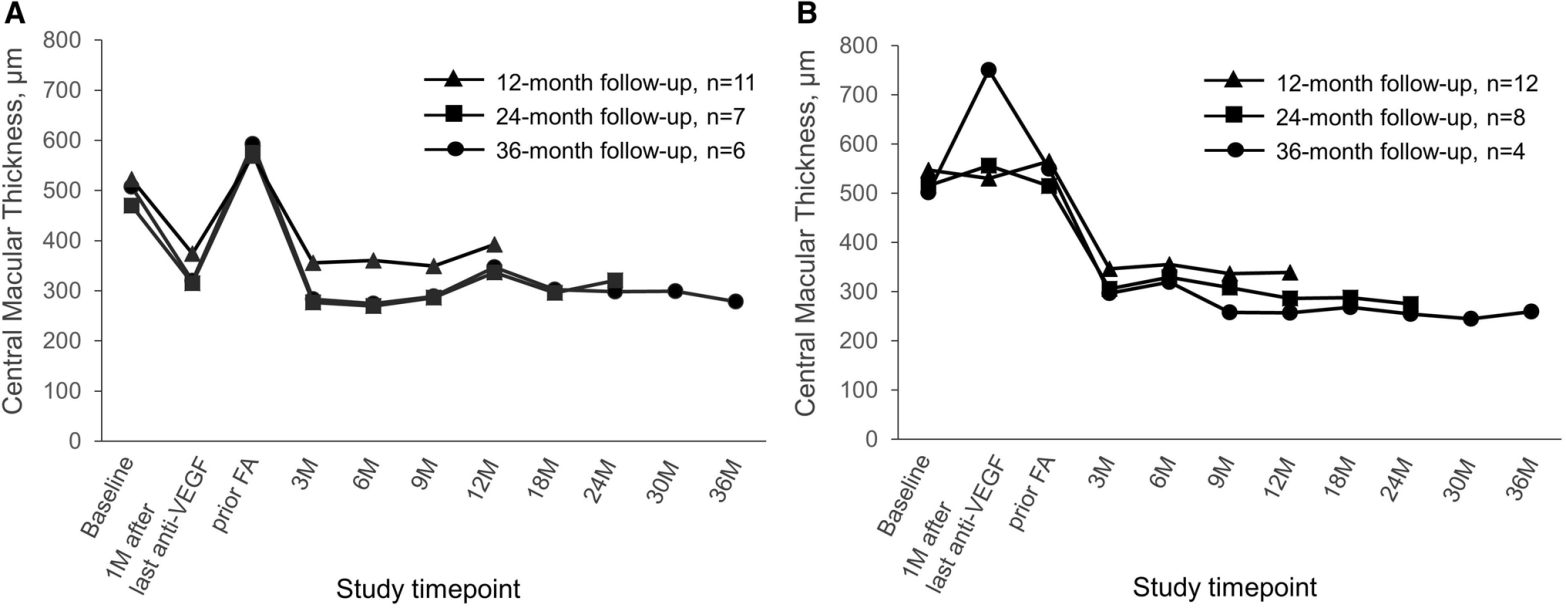
Mean change in CMT in pseudophakic eyes switched either directly (Group A; Panel A) or indirectly (Group B; Panel B) from anti-VEGF therapy to FAc implant. *CMT* central macular thickness, *FAc* fluocinolone acetonide, *VEGF* vascular endothelial growth factor

Overall in groups A and B, BCVA dropped around month 9 and improved from month 18 (Fig. 1). Stratification for lens status revealed that in eyes that were pseudophakic before FAc injection, BCVA and CMT improved significantly (*P* < 0.05) at all time points in Groups A and B (Figs. 3, 4, Table SD1 and SD2), supporting the impact of cataract progression on BCVA. For pseudophakic eyes in Group A, mean BCVA improvement at the end of follow-up was + 9.4 letters, + 14.4 letters and + 11.1 letters in the 12-, 24- and 36-month cohorts, respectively. For pseudophakic eyes in Group B, BCVA increased by + 7.1 letters, + 11.0 letters and + 11.3 letters in the 12-, 24- and 36-month cohorts, respectively, by the end of the study.

CMT also decreased in the pseudophakic eyes after FAc injection (Fig. 4 and Table SD2) with a significant (*P* < 0.05) improvement of 315.0 µm and 290.2 µm in Groups A and B, respectively, at Month 36.

### Additional treatments

Nine patients in Group A (37.5%) received additional intravitreal DME therapy after FAc injection as follows: additional dexamethasone implants (mean 1.7 ± 1.0) in three patients (12.5%); additional anti-VEGF injections (mean 2.2 ± 0.8) in four patients (16.7%); an additional FAc implant (24 months after first implant) in one patient (4.2%); and an additional dexamethasone implant + anti-VEGF injection in one patient (4.2%). In Group B, nine patients (36.0%) received additional DME therapy as follows: additional dexamethasone implants (mean number 1.6 ± 0.7) in eight patients (32.0%) and one additional dexamethasone implant + two anti-VEGF injections in one patient (4.0%).

The mean duration of FAc monotherapy (i.e., time from FAc implantation until additional treatments were given) was 22.7 ± 13.9 and 21.8 ± 9.6 months for Groups A and B, respectively. In Groups A and B, mean BCVA at the end of FAc monotherapy was 65.7 and 67.3 letters, an increase of 7.4 and 3.2 letters from pre-FAc levels, respectively. Mean CMT at the end of FAc monotherapy was 456.0 µm and 400.6 µm, a decrease of 147.7 µm and 169.0 µm from pre-FAc levels, respectively.

### Safety

In total, 14 patients (28.5%; 9 in Group A and 5 in Group B) had an IOP increase greater than > 21 mmHg after FAc treatment. In 13 eyes (26.5%; 6 in Group A and 7 in Group B), IOP had increased by more than > 10 mmHg compared with pre-FAc levels. These cases were successfully treated with IOP-lowering drops without needing surgery. Progression of cataract developed in 73.1% (19/26) of phakic eyes after FAc treatment. During the follow-up period, 26.9% (*n* = 7) of phakic eyes underwent phaco-emulsification (two eyes in Group A, five eyes in Group B).

## Discussion

In this single-center, real-world study, similar improvements in BCVA and CMT were seen in eyes treated with the FAc implant after insufficient anti-VEGF therapy, regardless of whether they first received a dexamethasone implant, or switched directly to the FAc implant. The significant improvements in BCVA and CMT were maintained for up to 36 months after FAc implantation in both those who switched directly after anti-VEGF or indirectly after a dexamethasone implant. Safety signals were consistent with corticosteroid class effects: small elevations in IOP were controlled with IOP-lowering medications when necessary. The rapidity, magnitude and duration of effect of the FAc implant seen in the current study are in line with findings from other real-world studies [19, 27–29].

In both Groups A and B, during the anti-VEGF treatment period, eyes experienced slight improvements (mean improvements of < 5 letters and < 100 µm) in both BCVA and CMT, respectively, but these were not sustained, despite each patient receiving a mean of 6.7 injections, and regressed to near baseline levels as soon as anti-VEGF therapy was stopped. Similarly, in Group B, during the dexamethasone treatment period, significant improvements in BCVA and CMT from pre-dexamethasone levels lasted for less than 4 months, despite some patients receiving more than 1 injection. In both Groups A and B, the improvements during FAc monotherapy were numerically greater than those achieved with intravitreal anti-VEGF injections. Hence, this study shows that a single FAc implant delivered long-lasting, significant functional and anatomical vision improvements, regardless of prior dexamethasone treatment, while eliminating the burden of repeated injections: a known barrier to delivering optimal patient care [12]. These findings are in line with previous reports and suggest that FAc may improve the standard and reduce the burden of care for this difficult-to-treat population [19, 27, 29, 30].

In both Groups A and B, there was a trend for BCVA to decrease between 9 and 18 months after FAc injection. This was more profound in eyes from Group A, whose BCVA dropped to lower than pre-FAc levels (evident in the 24- and 36-month cohorts). In the eyes previously treated with dexamethasone implants (Group B), the decrease was less obvious and presented between 9 and 12 months after FAc injection. As the same trend was not evident in pseudophakic eyes, we suggest that the BCVA decrease was due to the development of cataract which occurred in around three-quarters of phakic eyes. BCVA improvements were rapidly restored after cataract was removed and remained significantly higher than pre-FAc levels in all cohorts. These findings are consistent with the results from the FAME trials where a dip in mean BCVA was observed between months 9 and 18, followed by an improvement between months 18 and 24 following cataract surgery [11]. While cataract formation is undesirable, it is well known that steroid therapy that is still lasting at the time of cataract extraction helps to minimize inflammation and prevent pseudophakic cystoid macular edema (PCME): one of the most common vision-threatening complications of cataract surgery [31]. Recent reports suggest that FAc injection before cataract surgery may help to maintain stable macula, thus facilitating cataract surgery [32].

This study shows that functional and anatomical outcomes were similar between patients switched directly or indirectly to FAc after insufficient anti-VEGF therapy. Furthermore, it is known that DME eyes with lower baseline BCVA experience the greatest improvements in BCVA and eyes with good baseline BCVA have less potential to gain BCVA [33]. However, our study showed that FAc injection leads to significant improvements also in eyes with a good baseline BCVA (> 60 letters). Our findings suggest that for some patients who do not respond to the first-line therapies and who do not need prior steroid challenge, physicians may consider switching straight to FAc to avoid the need for repeated clinic visits and to achieve the best outcomes.

The limitations of this study included its retrospective design, which increases the potential for selection bias and confounding; the relatively small study sample size, which limits the generalizability of the results; a difference between both groups in terms of the history of macular laser treatment before the first injection of a steroid (i.e., DEX and/or the FAc implant) might have influenced the reported outcomes; and the lack of statistical analysis between Groups A and B, which limits the strength of the conclusions that can be drawn from the data.

## Conclusion

This study revealed the long-term safety and efficacy profile of the FAc intravitreal implant when used as second line for recurrent DME and shows that prior use of dexamethasone following an insufficient response to anti-VEGF therapy does not affect outcomes in patients subsequently treated with an FAc implant. Therefore, in patients with insufficient response to anti-VEGF treatment or long-term need of DME treatment, an early switch to FAc implant can be considered.

## Electronic supplementary material

Below is the link to the electronic supplementary material.

Supplementary material 1 (DOCX 14 kb)

Supplementary material 2 (DOCX 14 kb)

## References

[1] International Diabetes Federation (IDF) (2017) IDF Diabetes Atlas, 8th Edn. 2017. Available from: http://diabetesatlas.org/resources/2017-atlas.html. Accessed 9 Apr 2019

[2] Browning DJ, Stewart MW, Lee C (2018). Diabetic macular edema: evidence-based management. Indian J Ophthalmol.

[3] Lee R, Wong TY, Sabanayagam C (2015). Epidemiology of diabetic retinopathy, diabetic macular edema and related vision loss. Eye Vis (Lond).

[4] Cavan D, Makaroff L, da Rocha Fernandes J (2017). The diabetic retinopathy barometer study: global perspectives on access to and experiences of diabetic retinopathy screening and treatment. Diabetes Res Clin Pract.

[5] Gonder JR, Walker VM, Barbeau M (2014). Costs and quality of life in diabetic macular edema: canadian burden of diabetic macular edema observational study (C-REALITY). J Ophthalmol.

[6] Schmit-Eilenberger VK (2015). A novel intravitreal fluocinolone acetonide implant (Iluvien((R))) in the treatment of patients with chronic diabetic macular edema that is insufficiently responsive to other medical treatment options: a case series. Clin Ophthalmol.

[7] Ferris FL, Patz A (1984). Macular edema. A complication of diabetic retinopathy. Surv Ophthalmol.

[8] Royal College of Ophthalmologists (RCO) (2012) Diabetic Retinopathy Guidelines. 2012. Available from: https://www.rcophth.ac.uk/wp-content/uploads/2014/12/2013-SCI-301-FINAL-DR-GUIDELINES-DEC-2012-updated-July-2013.pdf. Accessed 9 Apr 2019

[9] International Council of Ophthalmology (ICO) (2017) ICO Guidelines for Diabetic Eye Care. 2017. http://www.icoph.org/downloads/ICOGuidelinesforDiabeticEyeCare.pdf. Accessed 9 Apr 2019

[10] Schmidt-Erfurth U, Garcia-Arumi J, Bandello F (2017). Guidelines for the management of diabetic macular edema by the european society of retina specialists (EURETINA). Ophthalmologica.

[11] Campochiaro PA, Brown DM, Pearson A (2012). Sustained delivery fluocinolone acetonide vitreous inserts provide benefit for at least 3 years in patients with diabetic macular edema. Ophthalmology.

[12] Eichenbaum DA, Buznego C, Weng CY (2018). When and how to incorporate steroids for persistent diabetic macular edema: a discussion of real-world treatment optimization strategies. Ophthalmic Surg Lasers Imaging Retina.

[13] Gillies MC, Sutter FK, Simpson JM (2006). Intravitreal triamcinolone for refractory diabetic macular edema: two-year results of a double-masked, placebo-controlled, randomized clinical trial. Ophthalmology.

[14] Martidis A, Duker JS, Greenberg PB (2002). Intravitreal triamcinolone for refractory diabetic macular edema. Ophthalmology.

[15] Busch C, Zur D, Fraser-Bell S (2018). Shall we stay, or shall we switch? Continued anti-VEGF therapy versus early switch to dexamethasone implant in refractory diabetic macular edema. Acta Diabetol.

[16] Allergan (2018) OZURDEX 700 micrograms intravitreal implant in applicator. 2018. Available at: https://www.medicines.org.uk/emc/product/5654. Accessed Jan 2018

[17] Alimera Sciences (2015) ILUVIEN 190 micrograms intravitreal implant in applicator. Available at: https://www.medicines.org.uk/emc/product/3061/smpc/print. Accessed Nov 2018

[18] Cunha-Vaz J, Ashton P, Iezzi R (2014). Sustained delivery fluocinolone acetonide vitreous implants: long-term benefit in patients with chronic diabetic macular edema. Ophthalmology.

[19] Pessoa B, Coelho J, Correia N (2018). Fluocinolone acetonide intravitreal implant 190 mug (ILUVIEN(R)) in vitrectomized versus nonvitrectomized eyes for the treatment of chronic diabetic macular edema. Ophthalmic Res.

[20] Khan Z, Kuriakose RK, Khan M (2017). Efficacy of the intravitreal sustained-release dexamethasone implant for diabetic macular edema refractory to anti-vascular endothelial growth factor therapy: meta-analysis and clinical implications. Ophthalmic Surg Lasers Imaging Retina.

[21] Fusi-Rubiano W, Blow RR, Lane M (2018). Iluvien (Fluocinolone Acetonide 0.19 mg Intravitreal Implant) in the treatment of diabetic macular edema: a review. Ophthalmol Ther.

[22] Alimera Sciences Ltd. ILUVIEN (fluocinolone acetonide) (2019) 190 micrograms intravitreal implant in applicator. Summary of Product Characteristics. October 2015

[23] World Medical Association (2018) WMA Declaration of Helsinki - Ethical Principles for Medical Research Involving Human Subjects 2013. Available at: https://www.wma.net/policies-post/wma-declaration-of-helsinki-ethical-principles-for-medical-research-involving-human-subjects/. Accessed 21 March 2018

[24] International Conference on Harmonisation of Technical Requirements for Registration of Pharmaceuticals for Human Use. *ICH Harmonised Tripartite Guideline*—*Guideline for Good Clinical Practice E6(R1)* 1996 [Available at: https://www.ich.org/fileadmin/Public_Web_Site/ICH_Products/Guidelines/Efficacy/E6/E6_R1_Guideline.pdf. Last Accessed 21 Mar 2018

[25] Novartis Pharmaceuticals UK Ltd. LUCENTIS (ranibizumab) 10 mg/ml solution for injection in pre-filled syringe. Summary of Product Characteristics. July 2018

[26] Allergan Pharmaceuticals Ireland. OZURDEX (dexamethasone) 700 micrograms intravitreal implant in applicator. Summary of Product Characteristics. November 2018

[27] Singh P, Chedid A, Deuchler SK (2018). The efficacy and safety outcomes of the 0.19 mg fluocinolone acetonide implant after prior treatment with the 0.7 mg dexamethasone implant in patients with diabetic macular edema. Int Med Case Rep J.

[28] Chakravarthy U, Taylor SR, Koch FHJ (2018). Changes in intraocular pressure after intravitreal fluocinolone acetonide (ILUVIEN): real-world experience in three European countries. Br J Ophthalmol.

[29] Mourtzoukos S (2017). The treatment of diabetic macular oedema (DMO) in UK real-life clinical practice with ILUVIEN (fluocinolone acetonide) - its impact on current clinical practice. Expert Review of Ophthalmology.

[30] Bailey C, Chakravarthy U, Lotery A (2017). Real-world experience with 0.2 mug/day fluocinolone acetonide intravitreal implant (ILUVIEN) in the United Kingdom. Eye (Lond).

[31] Grzybowski A, Kanclerz P (2019). The role of Steroids and NSAIDs in prevention and treatment of postsurgical cystoid macular edema. Curr Pharm Des.

[32] Ulbig M, Wehrman K, Maier M, editors. Second-line treatment with Iluvien for persistent pre-treated diabetic macular edema. ARVO Annual Meeting; Honolulu, Hawaii, US; April 29 - May 3 2018

[33] Alfaqawi F, Lip PL, Elsherbiny S (2017). Report of 12-months efficacy and safety of intravitreal fluocinolone acetonide implant for the treatment of chronic diabetic macular oedema: a real-world result in the United Kingdom. Eye (Lond).

