# Supplementary material for: Outcomes in diabetic macular edema switched directly or after a dexamethasone implant to a fluocinolone acetonide intravitreal implant following anti-VEGF treatment
Source: Acta Diabetol. 2019 Nov 20;57(4):469–78. doi: 10.1007/s00592-019-01439-x (PMC7093402; doi:10.1007/s00592-019-01439-x)
Supplement: Supplementary file 1 — Supplementary material 1 (DOCX 14 kb) [file 592_2019_1439_MOESM1_ESM.docx]

Table SD1. BCVA at each study timepoint in pseudophakic patients switched directly (Group A) or indirectly (Group B) from anti-VEGF therapy to FAc implant

| **BCVA at each study timepoint, ETDRS letters** | **Group A** | | | **Group B** | | |
| --- | --- | --- | --- | --- | --- | --- |
|  | **12-month cohort**  **(n=11)** | **24-month cohort**  **(n=7)** | **36-month cohort**  **(n=6)** | **12-month cohort**  **(n=12)** | **24-month cohort**  **(n=8)** | **36-month cohort**  **(n=4)** |
| Before FAc | 60.1 (10.4) | 58.6 (11.4) | 57.2 (11.8) | 63.8 (9.3) | 64.1 (9.1) | 60.5 (12.2) |
| 3 months after FAc  P value vs pre-FAc | 67.4 (11.4)  **0.004** | 67.6 (11.0)  **0.003** | 67.7 (12.1)  **<0.001** | 71.1 (11.8)  **0.011** | 75.8 (7.5)  **0.001** | 75.3 (10.4)  **0.008** |
| 6 months after FAc  P value vs pre-FAc | 68.6 (10.9)  **0.005** | 69.0 (12.5)  **0.004** | 69.5 (13.6)  **<0.001** | 70.4 (11.5)  **0.011** | 73.8 (10.4)  **0.008** | 71.0 (14.3)  0.094 |
| 9 months after FAc  P value vs pre-FAc | 68.4 (11.6)  **0.007** | 70.6 (12.2)  **<0.001** | 70.2 (13.3)  **<0.001** | 73.3 (9.2)  **<0.001** | 74.5 (9.0)  **0.003** | 73.0 (12.6)  **0.022** |
| 12 months after FAc  P value vs pre-FAc | 69.5 (10.7)  **0.005** | 71.4 (10.2)  **0.001** | 71.2 (11.2)  **0.001** | 70.9 (11.5)  **0.023** | 73.9 (11.2)  **0.033** | 74.5 (13.6)  **0.040** |
| 18 months after FAc  P value vs pre-FAc | –  – | 71.9 (12.3)  **<0.001** | 71.0 (13.2)  **0.001** | –  – | 71.4 (10.9)  **0.026** | 70.5 (14.8)  **0.016** |
| 24 months after FAc  P value vs pre-FAc | –  – | 73.0 (9.6)  **0.005** | 74.2 (9.9)  **0.001** | –  – | 75.1 (8.9)  **0.009** | 75.3 (10.3)  **0.009** |
| 30 months after FAc  P value vs pre-FAc | –  – | –  – | 72.3 (8.0)  **0.002** | –  – | –  – | 74.5 (9.6)  **0.002** |
| 36 months after FAc  P value vs pre-FAc | –  – | –  – | 68.3 (11.8)  **<0.001** | –  – | –  – | 71.8 (13.4)  **0.008** |

BCVA, best corrected visual acuity; ETDRS, Early Treatment Diabetic Retinopathy Study; FAc, fluocinolone acetonide; VEGF, vascular endothelial growth factor. All values are mean (SD).

Significant values are shown in bold.
