# Supplementary material for: Outcomes in diabetic macular edema switched directly or after a dexamethasone implant to a fluocinolone acetonide intravitreal implant following anti-VEGF treatment
Source: Acta Diabetol. 2019 Nov 20;57(4):469–78. doi: 10.1007/s00592-019-01439-x (PMC7093402; doi:10.1007/s00592-019-01439-x)
Supplement: Supplementary file 2 — Supplementary material 2 (DOCX 14 kb) [file 592_2019_1439_MOESM2_ESM.docx]

Table SD2. CMT at each study timepoint in pseudophakic patients switched directly (Group A) or indirectly (Group B) from anti-VEGF therapy to FAc implant

| **CMT at each study timepoint, ETDRS letters** | **Group A** | | | **Group B** | | |
| --- | --- | --- | --- | --- | --- | --- |
|  | **12-month cohort**  **(n=11)** | **24-month cohort**  **(n=7)** | **36-month cohort**  **(n=6)** | **12-month cohort**  **(n=12)** | **24-month cohort**  **(n=8)** | **36-month cohort**  **(n=4)** |
| Before FAc | 570.0 (166.6) | 575.4 (196.4) | 593.0 (209.0) | 564.8 (147.3) | 514.4 (106.1) | 550.0 (136.0) |
| 3 months after FAc  P value vs pre-FAc | 355.5 (137.6)  **0.002** | 276.9 (84.2)  **0.001** | 283.3 (90.4)  **0.003** | 346.7 (112.6)  **<0.001** | 305.9 (40.1)  **0.001** | 297.0 (37.8)  **0.014** |
| 6 months after FAc  P value vs pre-FAc | 360.2 (159.6)  **0.005** | 269.6 (91.4)  **0.001** | 274.0 (99.3)  **0.003** | 355.7 (127.4)  **0.001** | 329.8 (72.9)  **0.001** | 320.0 (87.2)  **0.005** |
| 9 months after FAc  P value vs pre-FAc | 349.2 (125.8)  **0.003** | 286.6 (81.5)  **0.002** | 289.0 (89.0)  **0.005** | 336.8 (147.8)  **0.004** | 307.9 (106.4)  **0.013** | 257.8 (94.2)  0.055 |
| 12 months after FAc  P value vs pre-FAc | 392.3 (156.3)  **0.005** | 336.6 (156.9)  **0.007** | 347.3 (169.0)  **0.017** | 339.1 (179.3)  **0.006** | 286.3 (81.3)  **0.005** | 257.0 (81.8)  0.051 |
| 18 months after FAc  P value vs pre-FAc | –  – | 295.7 (99.6)  **0.002** | 302.3 (107.4)  **0.004** | –  – | 288.1 (82.3)  **0.004** | 268.5 (94.0)  0.063 |
| 24 months after FAc  P value vs pre-FAc | –  – | 320.9 (117.0)  **0.013** | 298.5 (110.6)  **0.010** | –  – | 274.6 (68.3)  **0.002** | 254.8 (84.3)  **0.049** |
| 30 months after FAc  P value vs pre-FAc | –  – | –  – | 299.0 (123.0)  **0.012** | –  – | –  – | 244.8 (73.3)  **0.035** |
| 36 months after FAc  P value vs pre-FAc | –  – | –  – | 278.0 (82.4)  **0.007** | –  – | –  – | 259.8 (72.0)  **0.047** |

CMT, central macular thickness; FAc, fluocinolone acetonide; VEGF, vascular endothelial growth factor.

All values are mean (SD). Significant values are shown in bold.
